# Synthesis and Characterization of Novel Quaternary Ammonium Urethane-Dimethacrylate Monomers—A Pilot Study

**DOI:** 10.3390/ijms22168842

**Published:** 2021-08-17

**Authors:** Marta W. Chrószcz, Izabela M. Barszczewska-Rybarek

**Affiliations:** Department of Physical Chemistry and Technology of Polymers, Silesian University of Technology, 44-100 Gliwice, Poland; Marta.Chroszcz@polsl.pl

**Keywords:** urethane-dimethacrylates, quaternary ammonium compounds, photocurable dental resins

## Abstract

Six novel urethane-dimethacrylate analogues (QAUDMAs) were synthesized and characterized. They consisted of the 2,4,4,-trimethylhexamethylene diisocyanate (TMDI) core and two methacrylate-terminated wings containing quaternary ammonium groups substituted with alkyl chains of 8, 10, 12, 14, 16, or 18 carbon atoms. QAUDMAs, due to the presence of quaternary ammonium groups, may have possible antibacterial effects. Since they showed satisfactory physicochemical properties, they will be subjected to further research towards the development of dental composites with a capacity to reduce secondary caries. The synthesis of QAUDMAs included three stages: (i) transesterification of methyl methacrylate (MMA) with *N*-methyldiethanolamine (MDEA), (ii) *N*-alkylation of the tertiary amino group with alkyl bromide, and (iii) addition of TMDI to the intermediate achieved in the second stage. The formation of QAUDMAs was confirmed by ^1^H and ^13^C NMR. They were characterized for density (*d_m_*), viscosity (*η*), refractive index (*RI*), glass transition temperature (*T_g_*), polymerization shrinkage (*S*), and degree of conversion (*DC*). QAUDMAs were yellow, viscous resins (the *η* values ranged from 1.28 × 10^3^ to 1.39 × 10^4^ Pa·s, at 50 °C). Their *RI* ranged from 1.50 to 1.52, *T_g_* from −31 to −15 °C, *DC* from 53 to 78%, and *S* from 1.24 to 2.99%, which is appropriate for dental applications.

## 1. Introduction

Tooth decay constitutes a significant global health problem. Approximately 2.3 billion people worldwide, including 530 million children, today suffer from halitosis, toothache, tooth degradation, and tooth loss [1]. If left untreated, it may cause the inflammation of the tissue adjacent to the tooth and influence the pathogenesis of systemic diseases, such as rheumatoid arthritis, diabetes mellitus, and cardiovascular disease [2,3].

Tooth decay, also called dental caries, is caused by food debris metabolism of bacteria that form a biofilm on all mouth surfaces, predominantly on the tongue and teeth. The resulting acid causes demineralization of the hard tissues and subsequent cavity formation [3]. The form and function of damaged teeth can be restored by various treatments, among which dimethacrylate-based composite restorative materials are the most commonly used [4]. However, they do not protect against recurrent caries due to a greater tendency to dental plaque accumulation compared to enamel, or even other types of restoration materials [5]. Moreover, their hardening is accompanied by marginal gap formation, due to polymerization shrinkage, creating an environment that is favorable for bacterial growth [6]. The general strategy for this problem aims to design dental materials with antibacterial properties. The simplest solution is to admix microbiologically active particles, such as ZnO, TiO_2_, Au, Ag, antibiotics, chlorhexidine, etc. [7,8]. However, they tend to leach and irritate human tissues. The chemical modification of the composite matrix with monomers of antibacterial activity could result in a more stable material [9]. The application of quaternary ammonium methacrylate monomers (QAMs) offers a promising alternative. The quaternary ammonium group provides biocidal activity [10], whereas methacrylate groups provide covalent anchorage of a monomer to a composite matrix.

Several QAMs which show strong antibacterial effects are described in the literature [11,12,13,14,15,16,17]. In most cases, these are monomethacrylates (mono-QAMs). However, due to the presence of only one methacrylate group, they decrease the crosslink density of the composite matrix, resulting in increased monomer leaching [14,16,17] and mechanical weakening of the composite [15]. Therefore, they can only be added at low concentrations, which are insufficient to provide the composite with satisfactory antibacterial properties. For example, Ebi et al. reported that the maximum amount of 12-methacryloyloxydodecylpyridinium bromide, incorporated into a monomer composition (in the matrix of resin composites) should be limited to less than 0.4 wt.% [14].

Quaternary ammonium dimethacrylates (di-QAMs) are believed to be void of the abovementioned flaws of mono-QAMs. Two methacrylate groups provide a similar probability of di-QAM incorporation into a crosslinked composite matrix to that of the common dental monomers, such as bisphenol A glycerolate dimethacrylate (Bis-GMA), di-2-methacryloxyethyl 2,2,4-trimethylhexamethylenedicarbamate, commonly called the urethane-dimethacrylate monomer (UDMA), and triethylene glycol dimethacrylate (TEGDMA). The di-QAMs described in the literature include 2-methacryloxylethyl dodecyl and hexadecyl methyl ammonium bromides (respectively, MAE-DB and MAE-HB) [18], bis(2-methacryloyloxyethyl)dimethylammonium bromide (IDMA-1) [19], *N,N*′-bis[2 -(methacryloyloxy)ethyl]-*N,N,N′,N*′-tetramethyl-*N,N′*-butanediyl-, and hexanediyl-diammonium bromides (respectively, DMBB and DMBH) [20]. Each of them triggered the antibacterial effect of copolymers with common dental dimethacrylates. Antibacterial action against *Streptococcus mutans*—the most common human oral bacterium—was observed for the Bis-GMA/TEGDMA 75/25 wt.% polymer enriched with 10 wt.% of MAE-HB [21]; Bis-GMA/TEGDMA 50/50 wt.% polymer enriched with 10, 20, and 30 wt.% of IDMA-1 [19]; and commercially available adhesive resin (Tetric N-Bond) enriched with 1 wt.% of DMBB and DMBH [20].

The abovementioned monomers have rather simple fully aliphatic chemical structures that do not lend themselves well for use as Bis-GMA and UDMA replacements; thus, the QAM-modified polymers were probably not often tested for mechanical properties [19,20]. The literature provides only a few examples of QAMs with more advanced structures. Makvandi et al. achieved the Bis-GMA quaternary ammonium analogue (QABGMA) with two quaternary ammonium groups [16]. The addition of 5 to 15 wt.% QABGMA into the Bis-GMA/TEGDMA 50/50 wt.% polymer resulted in good antibacterial activity against various bacteria strains (including *S. mutans*), which increased as the QABGMA content increased. Liang et al. synthesized a series of fully aliphatic urethane-dimethacrylates with one central quaternary ammonium group substituted with aliphatic chains of 12 to 18 carbon atoms—*N,N*-bis[2-(3-(methacryloyloxy)propanamido)ethyl]-*N*-methylalkyl ammonium bromides (IQM) [22]. The addition of 10 to 20 wt.% IQM into the Bis-GMA/TEGDMA 50/50 wt.% composition resulted in polymers of good antibacterial activity against *S. mutans* and an increased degree of conversion, but a decreased flexural strength and modulus. In another study, Liang et al. synthesized the urethane-dimethacrylate with one central quaternary ammonium group, substituted with aliphatic chains of 12 to 18 carbon atoms and arms having two urethane linkages separated by the isophorone diisocyanate moiety (UDMQA) [23]. The UDMQA/TEGDMA 50/50 wt.% polymers showed satisfactory antibacterial activity against *S. mutans*, a high degree of conversion, and low polymerization shrinkage. However, their flexural strength, modulus, water sorption, and water solubility worsened [24].

**Scheme 1:**
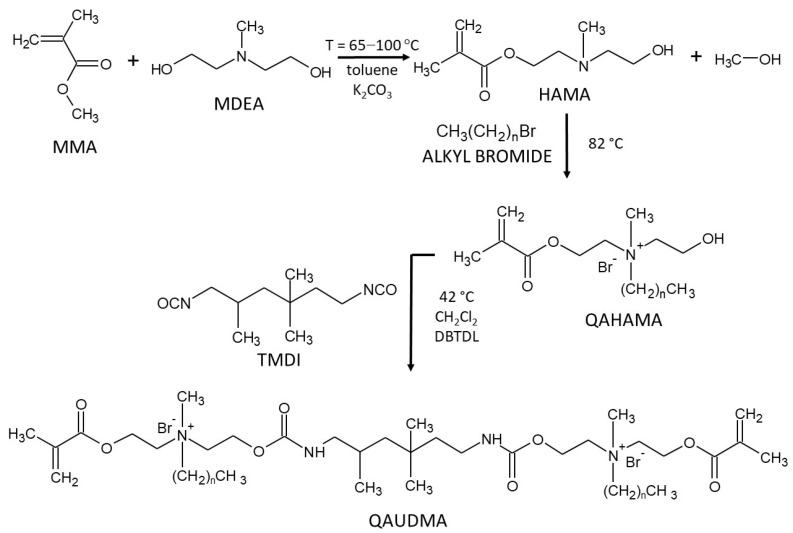
The synthesis route of the QAUDMA resins (*n* = 7–17).

To sum up, QAMs offer great potential for developing a new class of antimicrobial dental restorative materials, having the ability to reduce secondary caries and post-reconstructive inflammations. The literature shows that the dimethacrylate polymers enriched with QAMs have high antibacterial efficiency. However, their physicochemical and mechanical properties usually deteriorated. Therefore, studies on the development of new QAMs are still sought after. This study aimed at the synthesis and characterization of six novel TMDI-based QAMs (Scheme 1), being the UDMA analogues with possible antibacterial activity. The influence of the *N*-alkyl substituent, having from 8 to 18 carbon atoms, on the monomer density (*d_m_*), viscosity (*η*), refractive index (*RI*), glass transition temperature (*T_g_*), polymerization shrinkage (*S*), and degree of conversion (*DC*) was determined. This is a pilot study, and it will be continued by testing the QAUDMA-based dimethacrylate polymers for their antibacterial, physicochemical, and mechanical properties. The combination of two quaternary ammonium groups and two methacrylate groups in the QAUDMAs’ structures gives rise to the hypothesis that materials with adequate physicomechanical properties and a strong long-lasting antibacterial effect can be achieved at lower concentrations compared to QAMs known from the literature.

## 2. Results

Scheme 1 shows the chemical structures of all intermediate products and QAUDMAs. Their achievement was confirmed by ^1^H and ^13^C NMR spectroscopy.

**Figure 1:**
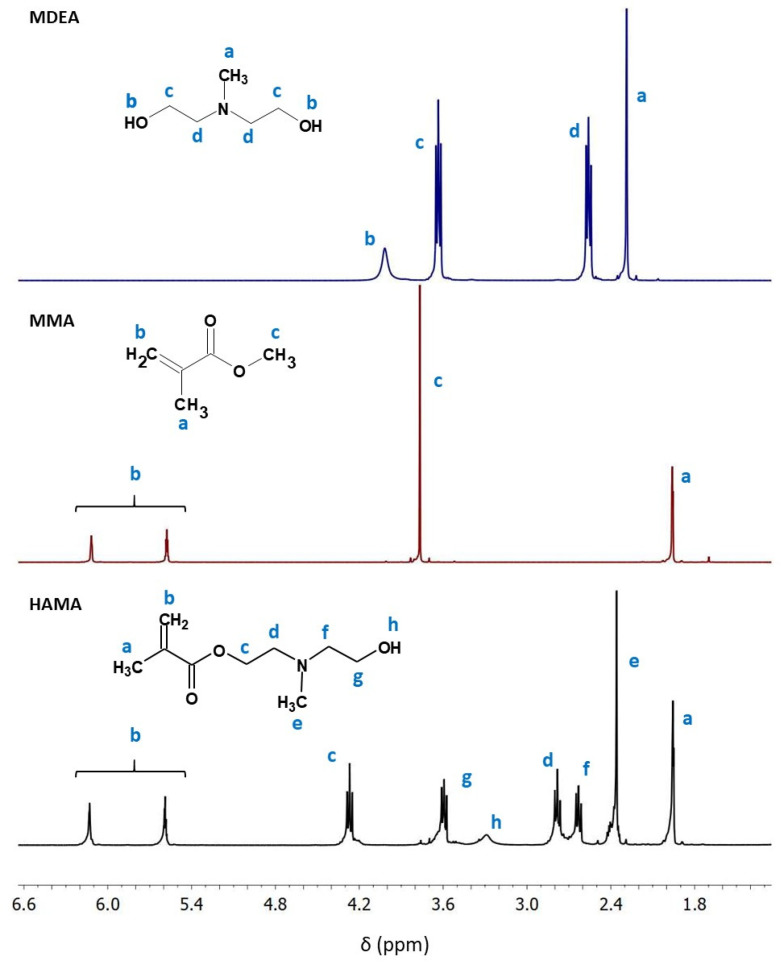
^1^H NMR spectra of MDEA, MMA, and HAMA.

^1^H NMR spectrum of HAMA (Figure 1) shows the disappearance of the peak from the -COOCH_3_ MMA protons (3.75 ppm, s, 3H). Each of the two signals in the MDEA spectrum, namely -CH_2_OH and -CH_2_N<, were split into two signals in the HAMA spectrum, respectively, -COOCH_2_- (4.27 ppm, t, 2H) and -CH_2_OH (3.62 ppm, t, 2H); and -COOCH_2_CH_2_N< (2.78 ppm, 2t, 4H) and >NCH_2_CH_2_OH (2.63 ppm, 2t, 4H). The broadened signal from the -OH group can also be seen (3.29 ppm, m).

**Figure 2:**
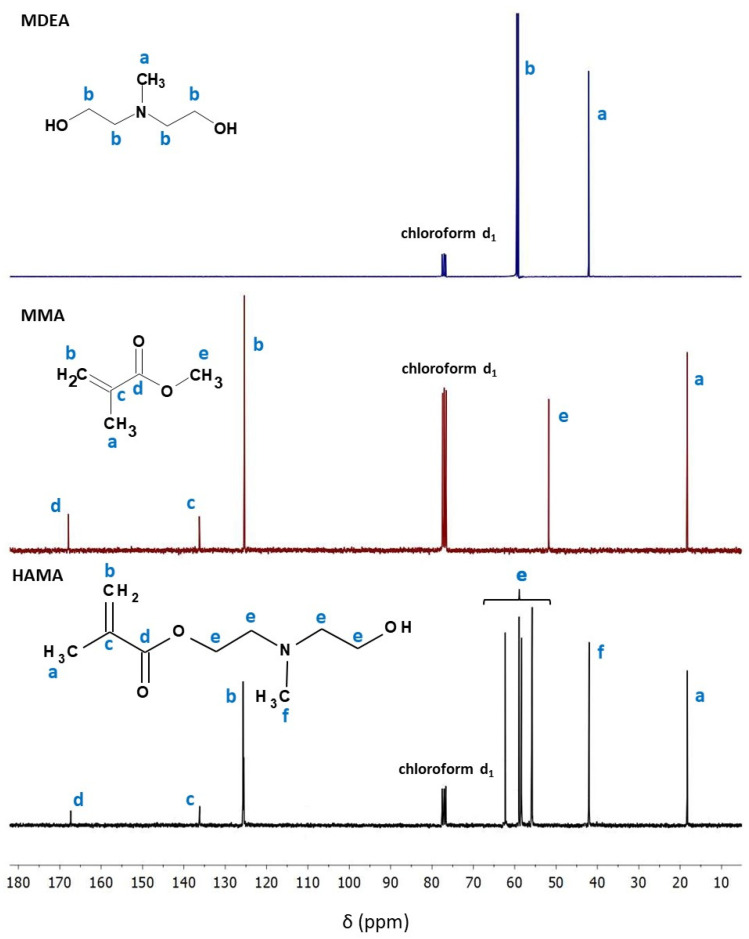
^13^C NMR spectra of MDEA, MMA, and HAMA.

The ^13^C NMR spectrum (Figure 2) shows the disappearance of the signal from the -COOCH_3_ MMA carbon (51 ppm). The two signals corresponding to the MDEA carbon atoms -CH_2_OH and -CH_2_N< were further split into two more signals each (55, 58, 59, and 62 ppm).

**Figure 3:**
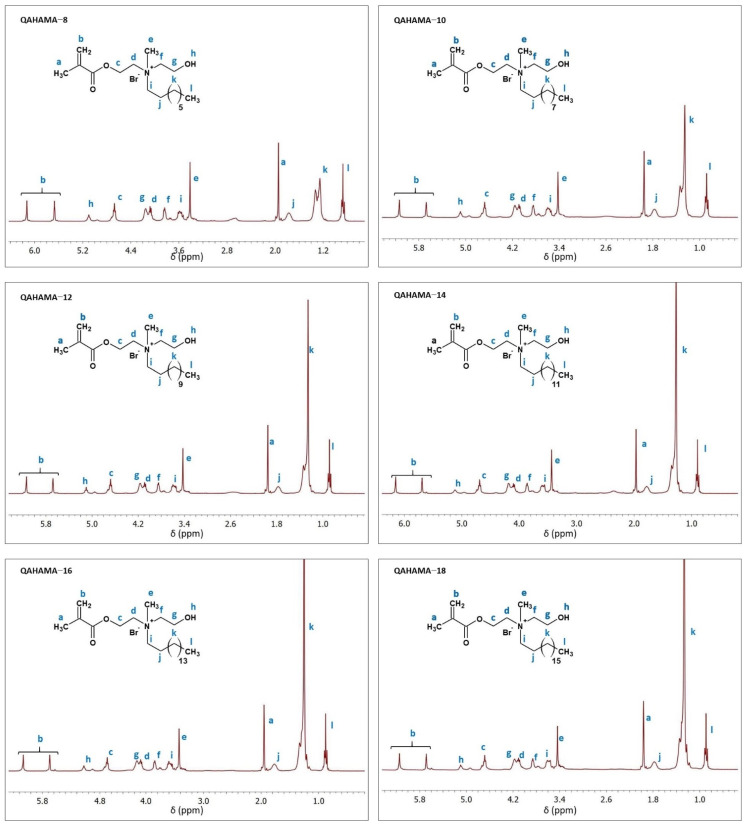
^1^H NMR spectra of QAHAMAs.

The ^1^H NMR spectra of QAHAMAs (Figure 3) confirmed the conversion of HAMA into quaternary ammonium salts (QAHAMAs). The signal from the >NCH_3_ protons (s, 3H) was de-shielded from 2.36 ppm (Figure 1) to 3.42 ppm, which is typical for the Menshutkin reaction, due to the formation of the >N^+^(CH_3_)- electron-withdrawing group [25]. The new signals, from the protons of the *N*-alkyl substituent, appeared in the ^1^H NMR spectra of QAHAMAs. They included protons from the following groups: -CH_3_ (0.87 ppm, t, 3H), oligomethylene chain neighboring the -CH_3_ group -(CH_2_)_n_CH_3_ (*n* = 5–15, depending on the length of the *N*-alkyl substituent) (1.17–1.43 ppm, m, from 10H to 30H, respectively for -C_8_H_17_ to -C_18_H_37_), second -CH_2_- from the ammonium nitrogen >N^+^(CH_3_)-CH_2_-CH_2_- (1.77 ppm, m, 2H), and -CH_2_- adjacent to the ammonium nitrogen >N^+^(CH_3_)-CH_2_- (3.59 ppm, m, 2H). In addition, signals from the remaining -CH_2_- groups were de-shielded to their location in the HAMA spectrum. Their new locations are as follows: -COOCH_2_- (4.67 ppm, m, 2H), -CH_2_OH (4.15 ppm, m, 2H), -COOCH_2_CH_2_N^+^(CH_3_)< (4.06 ppm, m, 2H), and >N^+^CH_2_CH_2_OH (3.85 ppm, m, 2H).

**Figure 4:**
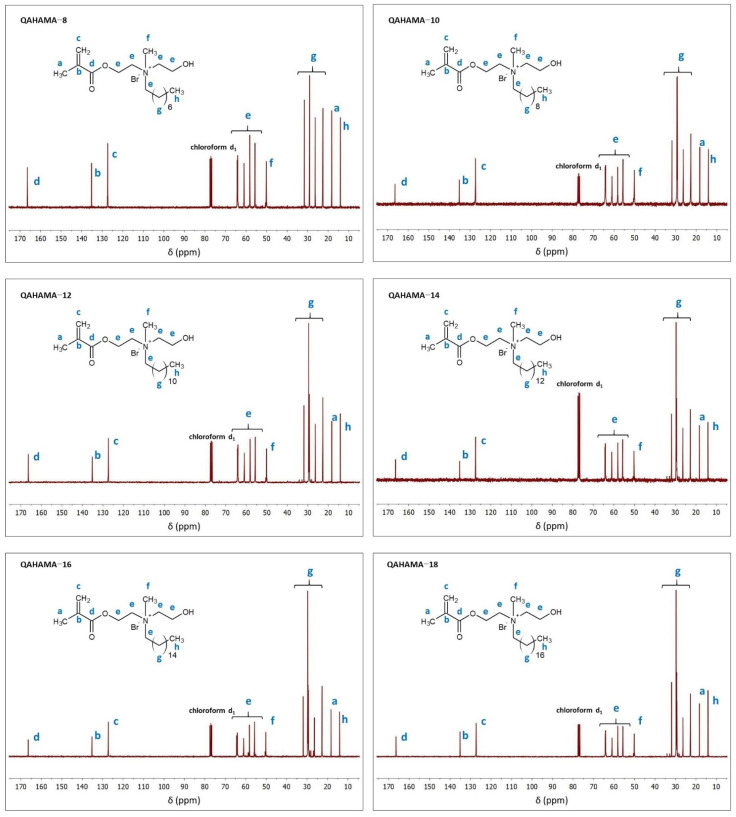
^13^C NMR spectra of QAHAMAs.

^13^C NMR spectra of QAHAMAs showed that the signal from the >NCH_3_ carbon in HAMA was de-shielded from 42 ppm (Figure 2) to 50 ppm (Figure 4) due to the Menshutkin reaction [25]. The *N*-alkyl substitution resulted in the appearance of new signals from carbon atoms of the following groups: -CH_3_ (14 ppm), oligomethylene chain neighboring to the -CH_3_ group -(CH_2_)_n_CH_3_ (*n* = 6–16, depending on the *N*-alkyl substituent length) (δ = 22, 26, 29, 31 ppm), and -CH_2_- adjacent the ammonium nitrogen >N^+^(CH_3_)-CH_2_- (64 ppm). Carbons of the remaining -CH_2_- group gave signals at 55, 58, 60, and 64 ppm.

**Figure 5:**
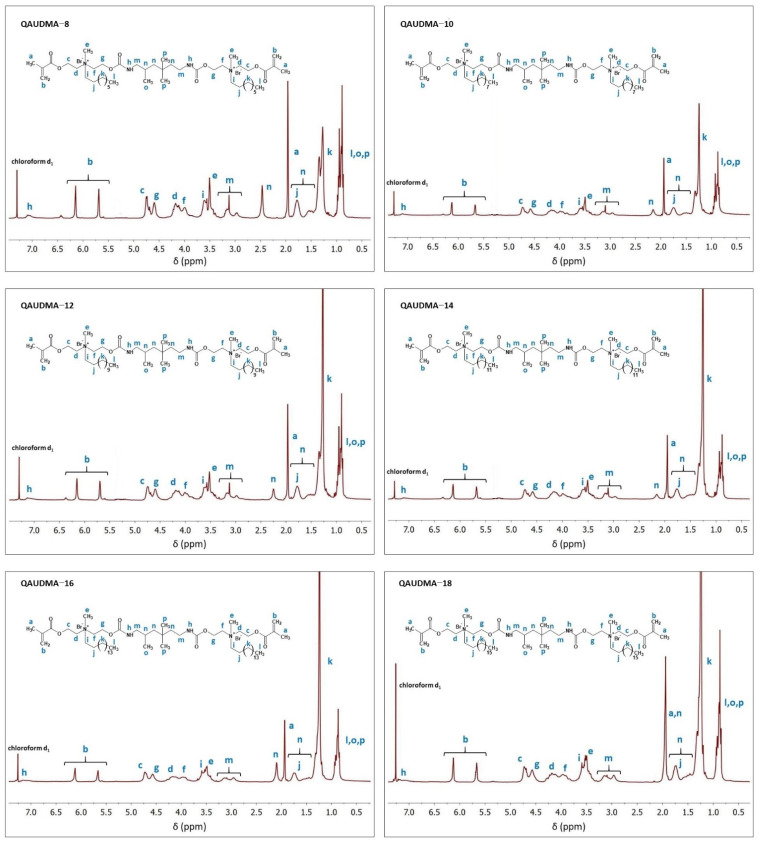
^1^H NMR spectra of QAUDMAs.

The NMR spectra of QAUDMAs confirmed the formation of the urethane bonds. On the ^1^H NMR spectra, the broadened signal from the proton of the urethane bond can be found at 7.18 ppm (m, 2H) (Figure 5). The signals from the protons of the TMDI -CH_2_- groups adjacent to the urethane bonds are located within the range of 2.85 to 3.25 ppm (m, 4H), whereas a series of multiplets from the remaining TMDI -CH_2_- and -CH< groups, which partially overlapped with the signals from -CH_2_- group of the N-alkyl substituent, are located within the range of 1.40 to 2.50 ppm. The signals from the TMDI -CH_3_ groups can be found within the range of 0.87 to 0.96 ppm and overlapped with the signal from the -CH_3_ group of the N-alkyl substituents.

**Figure 6:**
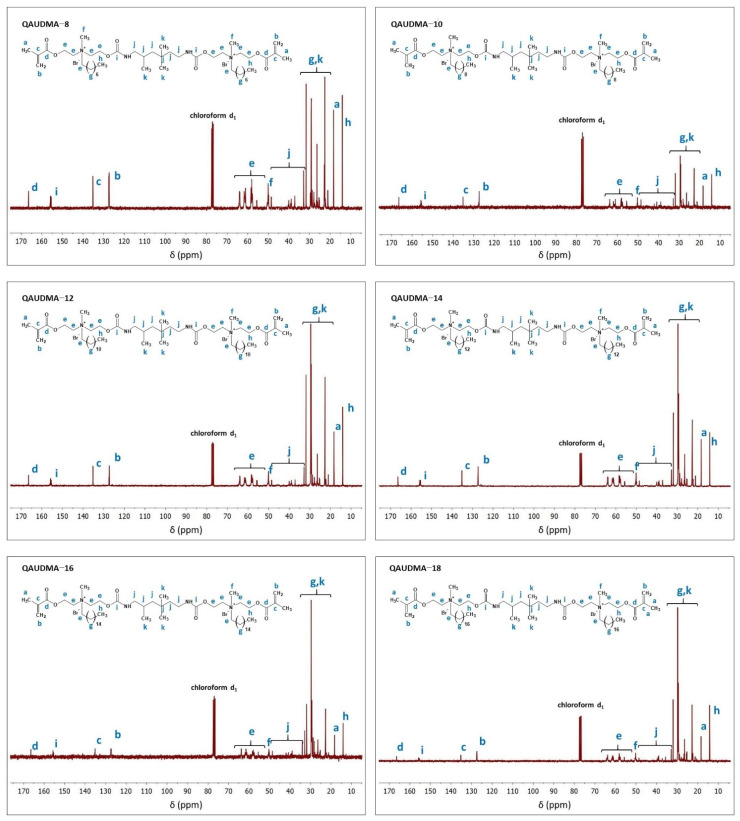
^13^C NMR spectra of QAUDMAs.

^13^C NMR spectra revealed the presence of the signal from the -NHCOO- group at 155 pm (Figure 6). Signals from the -CH_2_-, -CH<, and >C< groups of the TMDI core can be found at 32, 37, 39, 40, and 48 ppm, whereas the signals from the TMDI -CH_3_ groups were located at 21, 24, and 27 ppm.

**Figure 7:**
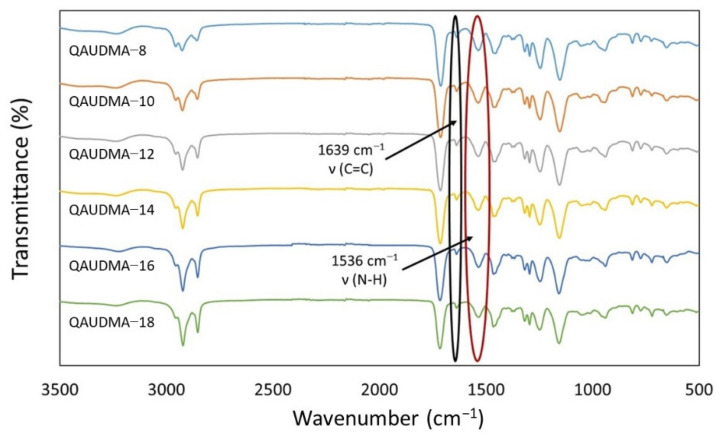
FTIR spectra of QAUDMAs.

The urethane bond formation was also confirmed by FTIR analysis (Figure 7). On the FTIR spectra of QAUDMAs, a band derived from the deformation vibrations of the N-H group can be found at 1536 cm^−1^ [26]. The stretching vibrations of the carbon–carbon double bond in the methacrylate group are observed at 1639 cm^−1^ [27,28].

**Figure 8:**
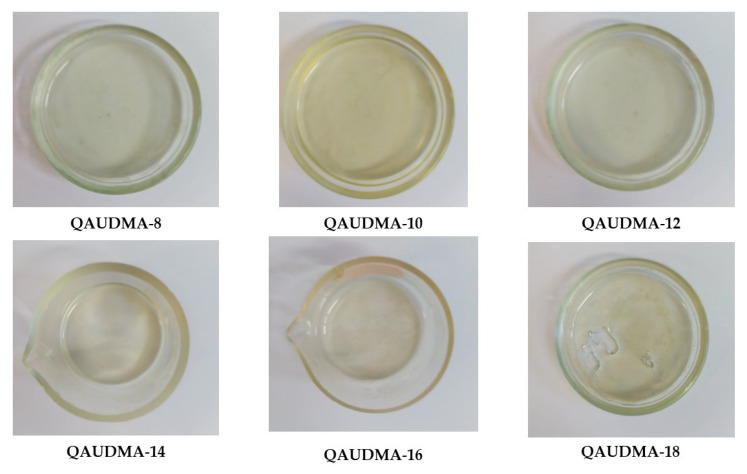
Photos of the obtained QAUDMAs.

**Figure 9:**
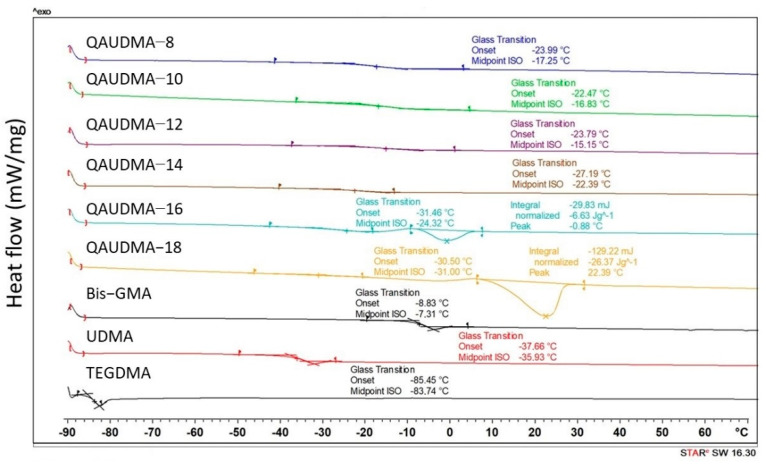
DSC thermograms of QAUDMAs, showing their *T_g_*.

In Table 1, the structural and physicochemical properties of QAUDMA monomers are summarized. The *MW* of QAUDMAs ranged from 970 to 1250 g/mol and increased with the increasing length of the *N*-alkyl substituent. The concentration of double bonds (*x_DB_*), which is equal to the concentration of urethane bonds (*x_UB_*), ranged from 1.60 to 2.06 mol/kg and decreased with the increasing length of the *N*-alkyl substituent. QAUDMAs were highly viscous resins with a slight yellow color (Figure 8). Their *RI* ranged from 1.5161 to 1.5003 and decreased as the *N*-alkyl substituent length increased. Their *η*, measured at 50 °C, ranged from 1.28 × 10^3^ to 1.39 × 10^4^ Pa·s. QAUDMA-10 and QAUDMA-12 revealed a distinct increase in the *η* value in respect to QAUDMA-8, and, hence, they were characterized by the highest viscosity. Further extension of the *N*-alkyl substituent caused a decrease in the *η* value, which achieved 3.33 × 10^3^ Pa·s for QAUDMA-18. The *T_g_* of QAUDMAs ranged from −31.01 to −15.15 °C (Figure 9). QAUDMA-8, having the shortest *N*-alkyl substituent, had the *T_g_* of −17.25 °C. It increased at the beginning, along with the increasing *N*-alkyl substituent length, and achieved the highest value for QAUDMA-12. Further lengthening of the *N*-alkyl substituent resulted in a *T_g_* decrease, and the lowest *T_g_* was recorded for QAUDMA-18. The *d_m_* of QAUDMAs ranged from 1.070 to 1.199 g/cm^3^ and decreased as the *N*-alkyl substituent length increased.

**Figure 10:**
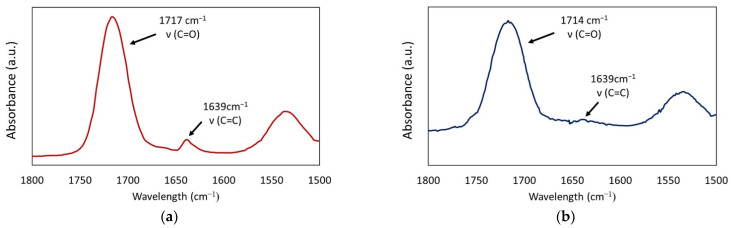
The representative FTIR spectra of the QAUDMA-16 monomer in its uncured (**a**) and cured (**b**) forms.

**Figure 11:**
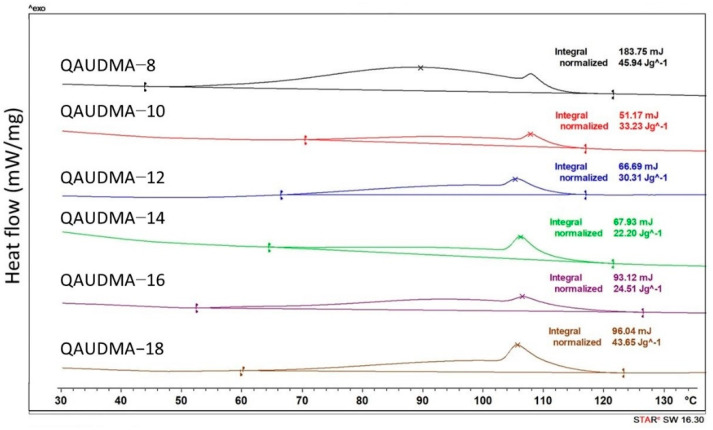
DSC thermograms of the QAUDMA polymers showing the post-polymerization peaks. On that basis, the *DC_DSC_* was calculated.

In Table 2, the results for *d_p_*, *S*, and *DC* are summarized. The *d_p_* ranged from 1.086 to 1.214 g/cm^3^ and decreased as the *N*-alkyl substituent length increased. The *S* ranged from 1.24 to 2.99%. Its values initially increased with the increasing length of the *N*-alkyl substituent length and achieved the highest value for QAUDMA-14. Further lengthening of the *N*-alkyl substituent caused a decrease in the *S* values. *S_theor_* values of QAUDMAs were also calculated. They ranged from 3.85 to 5.56% and decreased as the *N*-alkyl substituent length increased. The *DC* was determined by two methods, FTIR and DSC. As the QAUDMAs are fully aliphatic, the FTIR analysis was performed with the use of the carbonyl internal standard (Figure 10). The *DC_IR_* values were compared with those achieved from the DSC analysis (Figure 11), which was based on the measurement of post-polymerization heat. Both techniques gave similar results. The *DC_IR_* ranged from 53.37 to 79.42%, and *DC_DSC_* ranged from 52.83 to 78.12%. The *DC* increased with the lengthening of the *N*-alkyl substituent from 8 to 14 carbon atoms (QAUDMA-14 was characterized by the highest *DC*) and then decreased, reaching the lowest value for QAUDMA-18—having the longest *N*-alkyl substituent.

## 3. Discussion

In this study, a series of six quaternary ammonium UDMA analogues were successfully synthesized, as confirmed by ^1^H NMR, ^13^C NMR, and FTIR analyses (Figure 1, Figure 2, Figure 3, Figure 4, Figure 5, Figure 6 and Figure 7). They consisted of two methacrylate-terminated wings linked to the TMDI core via urethane bonds. In addition, the quaternary ammonium group was located in each wing. This objective was achieved in the three-stage route (Scheme 1): (i) transesterification of MMA, with MDEA resulting in HAMA formation; (ii) conversion of HAMA tertiary amine into the QAHAMA quaternary ammonium salt through the reaction with alkyl bromides, containing 8, 10, 12, 14, 16, and 18 carbon atoms; and (iii) addition of QAHAMA to TMDI.

As can be seen from Table 1, the novel QAUDMAs were characterized by relatively high molecular weights. Compared to Bis-GMA and UDMA, their *MWs* were about two times higher, whereas, compared to TEGDMA, they were almost four times higher. It was reflected in a lower *x_DB_*, as compared to Bis-GMA, UDMA, and TEGDMA. It can have both beneficial and adverse effects on the composite matrix properties. Respectively, low *x_DB_* typically results in low polymerization shrinkage but causes greater remoteness of junction points in a crosslinked polymer, causing a decrease in the crosslink density [28]. QAUDMAs also contained two urethane bonds, whose concentration (*x_UB_*) decreased with the increasing length of the *N*-alkyl substituent. The presence of the -NHCOO- linkages can be a valuable quality for the polymer properties because they are responsible for the physical crosslinking [28]. Their *RI* values were within the range of 1.46 to 1.55, indicating transparency similar to that of tooth enamel [29]. All QAUDMAs took the liquid form, which is crucial for the formulation process. However, they were characterized by significantly higher viscosities than common dental monomers. Bis-GMA revealed the *η* value of 8.36 Pa·s at 50 °C, which is three and four orders of magnitude lower compared to the studied QAUDMAs. This indicates that, if QAUDMAs were applied as components of dental dimethacrylate materials, the use of the reactive diluent would be necessary (all QAUDMAs were soluble in TEGDMA). The *η* varied according to the *N*-alkyl substituent length. Its value significantly increased at the beginning and then decreased. As a result, QAUDMA-10 and QAUDMA-12 were characterized by the highest viscosity. It indicates that the initial extension of the *N*-alkyl substituent caused a reduction in the molecular mobility of QAUDMAs. Its further extension probably had a plasticizing effect, which could cause an increase in molecular mobility and the weakening of intermolecular interactions. The *T_g_* of QAUDMAs was determined to verify the above assumption (Figure 9). The pattern observed for the *T_g_* values matched that found for viscosity values, which confirmed that the molecular interactions between monomer molecules having from 8 to 12 carbon atoms in the *N*-alkyl substituents were stronger than between those having the *N*-alkyl substituents of at least 14 carbon atoms. It could also suggest that the *N*-alkyl substituents in lengths from 8 to 12 carbon atoms can more tightly pack than the longer ones. Compared to dental dimethacrylates, all QAUDMAs were characterized by lower *T_g_* than Bis-GMA, but higher than UDMA and TEGDMA. It indicates that QAUDMAs are characterized by greater molecular mobility than Bis-GMA, and, therefore, they could be expected to polymerize to *DCs* higher than Bis-GMA (its low *DC* results from the limitations caused by its stiff molecular structure and strong hydrogen bonds [30,31]). The measurements of the *d_m_* of QAUDMAs confirmed that, the longer the *N*-alkyl substituent, the lower the ability of the QAUDMA molecule to pack tightly (*d_m_* values decreased as the *N*-alkyl substituent length increased). Compared to dental dimethacrylates, QAUDMAs having from 8 to 12 carbon atoms in the *N*-alkyl substituents were characterized by a higher *d_m_* than Bis-GMA, and those having 14 and 16 carbon atoms in the *N*-alkyl substituent revealed *d_m_* values which approached those of UDMA. QAUDMA-18 had the same *d_m_* as TEGDMA. It shows that the fully aliphatic character and presence of urethane bonds in the QAUDMAs’ structures provide high molecular elasticity that enables us to achieve tight, tangled conformations and often results in denser packing compared to dental dimethacrylates.

As can be seen from Table 2, the *d_p_* values were lower than the corresponding *d_m_* values, which is obvious due to polymerization shrinkage. Compared to dental dimethacrylates, QAUDMAs were usually characterized by significantly lower *S*, except QAUDMA-14. The *S* value of the latter was higher than that of UDMA, with a difference of about 28%. *S_theor_* was also calculated. Its values for QAUDMAs were always lower than half those of dental dimethacrylates. It could be an advantage for future-designed dental materials with low polymerization shrinkage. The detailed comparison of the *S* and *S_theor_* values within the QAUDMA series led to the observation that an increase of the N-alkyl substituent length from 8 to 14 carbon atoms caused an increase in *S* values, but a decrease in the *S_theor_* values. Both parameters showed a decreasing trend only from QAUDMA-16. This discrepancy pointed to the variability in the *DC*, which was determined by FTIR (Figure 10) and DSC (Figure 11). The observed pattern coincided well with the results for the polymerization shrinkage. The *DC*, as well as *S*, increased from QAUDMA-8 to QAUDMA-14, indicating that the *DC* was a decisive factor in the volumetric contraction. Other important factors influencing *S*, such as differences in monomer molecular dimensions, shape, and hydrophilicity [32], appeared to be less significant. Interestingly, the results for the *DC* did not correlate to the results for the *η* and *T_g_*, which are commonly regarded as factors that determine the *DC* in dimethacrylate systems [30]. The lower the *η* and *T_g_*, the higher the *DC* [30]. In the studied series, QAUDMA-12, which was characterized by the highest *T_g_* and second-highest *η*, revealed the second-highest *DC*. QAUDMA-14, characterized by the highest *DC*, was the first monomer whose *η* and *T_g_* radically decreased due to the lengthening of the *N*-alkyl substituent. It implies that the presence of *N*-alkyl substituent with 14 carbon atoms provides the monomer with optimum molecular elasticity and strength of intermolecular interaction, leading to the highest *DC*. Regarding the requirements for structural quality of dental dimethacrylate polymer networks, the *DC* of all studied QAUDMAs was satisfactorily high. Its values were always higher than 50%, which is conducive to the theory of dimethacrylate polymer networks. They were also usually higher than 55% (except for QAUDMA-18, having a *DC* of about 53%), which is requested by a clinical experience [28]. Compared to dental dimethacrylates, QAUDMAs were characterized by *DCs*, the most similar to that of UDMA.

## 4. Materials and Methods

### 4.1. Materials

Alkyl bromides, MDEA, and MMA were purchased from Acros Organics (Geel, Belgium). TMDI was purchased from Tokyo Chemical Industry (Tokyo, Japan). Camphorquinone (CQ), 2-dimethylaminoethyl methacrylate (DMAEMA), phenothiazine (PTZ), and tetramethylsilane (TMS) were purchased from Sigma-Aldrich (St. Louis, MO, USA). Dibutyltin dilaurate (DBTDL) was purchased from Fluka (Charlotte, NC, USA). Chloroform, methylene chloride, potassium carbonate, and toluene were purchased from POCH S.A. (Gliwice, Poland). All reagents were used as received.

### 4.2. Monomer Synthesis

#### 4.2.1. Synthesis of *N,N*-(2-Hydroxyethyl)methylaminoethyl Methacrylate (HAMA)

MMA (1 mol, 100.12 g), MDEA (0.67 mol, 79.85 g), K_2_CO_3_ 8 wt.%—a transesterification catalyst, PTZ 500 ppm—a polymerization inhibitor, and toluene (400 mL) were introduced into a 1000 mL round-bottom flask equipped with a standard laboratory distillation kit. The reaction mixture was heated by a combined hot-plate magnetic-stirrer device via an oil bath for 2.5 h when the temperature at the top of the column achieved 100 °C. K_2_CO_3_ was filtered, and the reaction mixture was washed with distilled water in a 2:1 volume ratio. The aqueous layers were extracted with chloroform in a 3:1 volume ratio. The chloroform layer was dried with MgSO_4_, and chloroform was removed with a rotary evaporator under reduced pressure (10 mbar). The crude product HAMA was distilled under a vacuum (2 mbar), taking boiling fraction at 120–130 °C. The final product yield was 19%.

#### 4.2.2. Synthesis of 2-(Methacryloyloxy)ethyl-2-hydroxyethylmethylalkylammonium Bromides (QAHAMAs)

HAMA (0.107 mol, 20.0 g), alkyl bromide (0.107 mol, from 20.66 to 35.67 g, depending on the alkyl chain length; see Table 3), and PTZ (500 ppm) were introduced into a 250 mL three-neck round-bottom flask equipped with a reflux condenser and thermometer. The reaction mixture was mixed with the mechanical stirrer and heated at 82 °C for 82 to 168 h (depending on the alkyl bromide; see Table 3) via an oil bath.

#### 4.2.3. Synthesis of Quaternary Ammonium Urethane-Dimethacrylates (QAUDMAs)

A 50 wt.% solution of QAHAMA (0.107 mol, from 40.66 to 55.67 g, depending on the *N*-alkyl chain length; see Table 4) in methylene chloride was introduced into a 250 mL three-neck round-bottom flask equipped with a reflux condenser, dropping funnel, and thermometer. DBTDL 0.03 wt.%, a catalyst, and PTZ 500 ppm were added into the same flask. The reaction mixture was heated to boiling point by a combined hot-plate magnetic-stirrer device via an oil bath. Then, the 50 wt.% solution of TMDI (0.054 mol, 11.24 g) in methylene chloride was added dropwise for 1 h, and the reaction mixture was refluxed for the next 3 h. Methylene chloride was then evaporated under a vacuum (firstly at 30 mbar, and then at 3 mbar), and QAUDMAs were achieved with 100% yield.

### 4.3. Nuclear Magnetic Resonance (NMR)

^1^H and ^13^C NMR spectra were recorded in the CDCl_3_ solutions, using the TMS internal standard, with, respectively, 128 and 40,000 scans, utilizing 300 MHz NMR spectrometer (UNITY/INOVA, Varian, Palo Alto, CA, USA).

### 4.4. Fourier Transform Infrared Spectroscopy (FTIR)

FTIR spectra were recorded with 128 scans at a resolution of 1 cm^−1^, utilizing a Spectrum Two (Perkin-Elmer, Walthma, MA, USA) spectrometer. Liquid substances were tested in the form of a thin layer placed between two KBr pellets. The polymers, achieved by photopolymerization with the CQ/DMAEMA 0.4/1 wt.% initiating system and UV–VIS lamp (Ultra Vitalux 300, Osram, Munich, Germany), were powdered and analyzed as KBr pellets. The degree of conversion *(DC_IR_*) was calculated according to the following equation:

(1) $$DC_{IR}\left(\%\right)=\left(1-\frac{{\left(\frac{A_{C=C}}{A_{C=O}}\right)}_{polymer}}{{\left(\frac{A_{C=C}}{A_{C=O}}\right)}_{monomer}}\right)\times 100,$$

where *A_C=C_* is the absorption intensity of the carbon–carbon double bond stretching vibrations at 1639 cm^−1^, and *A_C=O_* is the absorption intensity of the carbonyl group stretching vibrations at 1714 cm^−1^.

### 4.5. Differential Scanning Calorimetry (DSC)

DSC measurements were performed in the temperature range of −90 to 200 °C, with a heating rate of 10 K/min, in the air, utilizing the DSC 3 (Mettler Toledo, Greifensee, Switzerland) apparatus. The glass transition temperature (*T_g_*) was taken as the midpoint of the transition region. The degree of conversion (*DC_DSC_*) was determined from the post-curing heat of powdered QAUDMAs’ polymer samples admixed with BPO 0.5 wt.%. The polymers were previously achieved by photopolymerization with the CQ/DMAEMA 0.4/1 wt.% initiating system and a UV–VIS lamp (Ultra Vitalux 300, Osram, Munich, Germany). The *DC_DSC_* was calculated according to the following equation:

(2) $$DC_{DSC}\left(\%\right)=\left(1-\frac{MW\times \Delta H_{exp}}{f\times \Delta H_{pol}}\right)\times 100,$$

where *MW* is the QAUDMA molecular weight (g/mol), Δ*H_exp_* is the experimentally determined post-polymerization heat (kJ/g), *f* is the number of methacrylate groups in the QAUDMA molecule (*f* = 2), and Δ*H_pol_* is the enthalpy of the polymerization of 1 mole of methacrylate group (Δ*H_pol_* = 57.8 kJ/mol [31]).

### 4.6. Density and Polymerization Shrinkage

The monomer densities (*d_m_*) were determined with a pycnometer. The polymer densities (*d_p_*) were measured in water, utilizing an analytical balance with a 0.01 mg accuracy (XP balance, Mettler Toledo, Greifensee, Switzerland), equipped with a density determination kit. The polymerization shrinkage (*S*) was calculated according to the following equation:

(3) $$S\left(\%\right)=\left(1-\frac{d_{m}}{d_{p}}\right)\times 100,$$

where *d_m_* is the monomer density, and *d_p_* is the polymer density.

The theoretical polymerization shrinkage (*S_t_*) was calculated, assuming 100% conversion of the methacrylate groups, according to the following equation:

(4) $$S_{theor}\left(\%\right)=\left(\frac{f\times \Delta V\times d_{m}}{MW}\right)\times 100,$$

where *f* is the number of methacrylate groups in the QAUDMA molecule (*f* = 2), ∆*V* is the decrease in molar volume due to the polymerization of one methacrylate group (∆*V* = 22.5 cm^3^/mol [33]), and *MW* is the QAUDMA molecular weight.

### 4.7. Viscosity

The viscosity (*η*) was determined by utilizing Kinexus pro+ (Netzsch, Selb, Germany) rotational rheometer, with the use of a Peltier plate cartridge and a cone-plate measuring system with a gap resolution of 0.15 mm. The *η* of QAUDMA was measured at 50 °C, and that of Bis-GMA was at 25 and 50 °C, whereas that of UDMA was at 25 °C.

### 4.8. Refractive Index

The refractive index (*RI*) of monomers was determined by utilizing DR 6100T (Krüss Optronic, Germany) digital refractometer at 20 °C, according to ISO 489:1999 [34].

### 4.9. Statistical Analysis

Statistical analysis was performed with the Statistica 13.1 (TIBCO Software Inc., Palo Alto, CA, USA) software. Five samples were tested for each of the monomers and measurements. The results were expressed as average values and corresponding standard deviations (*SD*). The non-parametric Wilcoxon test with a significance level (*p*) of 0.05 was used to determine the statistical significance of the results.

## 5. Conclusions

New QAUDMA monomers, namely the UDMA analogues, can be successfully synthesized via a three-stage process, including (i) transesterification of MMA with MDEA, leading to HAMA formation; (ii) *N*-alkylation of the HAMA tertiary amino group with alkyl bromide, leading to QAHAMA formation; and (iii) addition of TMDI to QAHAMA. All of them were highly viscous resins, showing viscosities greater than Bis-GMA, UDMA, and TEGDMA. If they were used as components of dental composites, they would require the use of a reactive solvent, such as TEGDMA. QAUDMAs were characterized by low *S*, high *DC*, and *RI* required for applications in dental composites. This pilot study showed that QAUDMAs may be interesting components of dental dimethacrylate formulations with potential antibacterial properties. Further studies will focus on testing the mechanical, physicochemical, and biocidal properties of the QAUDMA-based copolymers.

## Figures and Tables

**Table 1:** The properties of QAUDMA monomers. Couples that revealed statistically significant (*p* < 0.05) results are marked with the letters.

Sample Name	*MW* (g/mol)	*x_DB_* = *x_UB_* (mol/kg)	*RI* ^1^	*η* (Pa·s) (50 °C)		*T_g_* (°C)		*d_m_* (g/cm^3^)	
				Avg.	SD	Avg.	SD	Avg.	SD
Bis-GMA	512	3.90	1.5493	1.14 × 10^3 2,a–f^	0.13 × 10^3^	−7.31 ^a–h^	0.76	1.15 ^a–h^	0.01
				8.36	0.2				
UDMA	470	4.25	1.4614	9.54 ^2^	0.02	−35.93 ^a,i–o^	1.24	1.09 ^a,i–l^	0.01
TEGDMA	286	6.99	1.4852	0.011 ^2^	-	−83.74 ^b,i,p–v^	1.56	1.07 ^b,m–o^	0.02
QAUDMA-8	970	2.06	1.5161	1.28 × 10^3 a,g,h–k^	0.28 × 10^3^	−17.25 ^c,j,p,w–z^	0.61	1.199 ^c,i,m,p–u^	0.01
QAUDMA-10	1026	1.95	1.5112	1.39 × 10^4 b,g,l–n^	0.14 × 10^4^	−16.38 ^d,k,r,a’–c’^	0.71	1.166 ^d,j,n,p,v–y^	0.01
QAUDMA-12	1082	1.85	1.508	1.14 × 10^4 c,h,o–r^	0.13 × 10^4^	−15.15 ^e,l,s,w,d’–f’^	1.03	1.129 ^e,k,o,r,v,z,a’,b’^	0.01
QAUDMA-14	1138	1.76	1.5054	3.18 × 10^3 d,i,l,o^	0.36 × 10^3^	−22.39 ^f,m,t,x,a’,d’,g’^	1.71	1.101 ^f,l,s,w,z,c’,d’^	0.01
QAUDMA-16	1194	1.68	1.5005	2.79 × 10^3 e,j,m,p^	0.39 × 10^3^	−24.32 ^g,n,u,y,b’,e’,h’^	1.65	1.085 ^g,t,x,a’,c’,d’^	0.02
QAUDMA-18	1250	1.60	1.5003	3.33 × 10^3 f,k,n,r^	0.32 × 10^3^	−31.01 ^h,o,v,z,c’,f’-h’^	0.92	1.070 ^h,u,y,b’^	0.01

^1^ Standard deviation for the refractive index was 0.0001 in every case. ^2^ As cited in Reference [27] and measured at 25 °C.

**Table 2:** The properties of QAUDMA polymers. Couples that revealed statistically significant results (*p* < 0.05) are highlighted with the letters.

Sample Name	*d_p_* (g/cm^3^)		*S_theor_* (%)	*S* (%)		*DC_IR_* (%)		*DC_DSC_* (%)	
	Avg.	SD		Avg.	SD	Avg.	SD	Avg.	SD
Bis-GMA	1.207 ^a–d^	0.058	10.11	4.72 ^a–h^	0.19	32.56 ^a–h^	0.92	- ^1^	- ^1^
UDMA	1.116 ^a,e–g^	0.054	10.65	2.33 ^a,i–m^	0.26	78.92 ^a,i–n^	1.33	- ^1^	- ^1^
TEGDMA	1.197 ^e,h–k^	0.021	17.12	10.61 ^b,i,n–t^	0.20	84.23 ^b,i,o–u^	0.91	- ^1^	- ^1^
QAUDMA-8	1.214 ^f,l–o^	0.057	5.56	1.24 ^c,j,n,u–y^	0.07	60.39 ^c,j,o,v–z^	1.41	61.36 ^a–d^	3.9
QAUDMA-10	1.186 ^g,p,r^	0.038	5.11	1.69 ^d,k,o,u,z,a’,b’^	0.16	71.76 ^d,k,p,v,a’–c’^	2.31	70.76 ^a,e,f^	3.54
QAUDMA-12	1.155 ^h,l,s,t^	0.016	4.69	2.25 ^e,p,v,c’,d’^	0.22	73.35 ^e,l,r,w,d’,e’^	2.49	71.64 ^b,g,h^	4.9
QAUDMA-14	1.135 ^b,i,m,u^	0.023	4.35	2.99 ^f,l,r,w,z,c’,e’,f’^	0.37	79.42 ^f,s,x,a’,d’,f’,g’^	1.94	78.12 ^c,e,g,i^	2.59
QAUDMA-16	1.107 ^c,j,n,p,s^	0.019	4.09	1.99 ^g,s,x,a’,e’,g’^	0.10	74.79 ^g,m,t,y,b’,f’,h’^	2.03	74.27 ^d,j^	2.95
QAUDMA-18	1.086 ^d,k,o,r,t,u^	0.009	3.85	1.47 ^h,m,t,y,b’,d’,f’,g’^	0.04	53.37 ^h,n,u,z,c’,e’,g’,h’^	2.67	52.83 ^f,h,i,j^	2.77

^1^ Was not measured.

**Table 3:** Weights of alkyl bromides and reaction times in the *N*-alkylation stage (formation of QAHAMAs).

Reagent	Mass (g)	Reaction Time (h)
1-bromooctane	20.66	82
1-bromodecane	23.66	
1-bromododecane	26.67	
1-bromotetradecane	29.67	
1-bromohexadecane	32.67	168
1-bromooctadecane	35.67	

**Table 4:** Weights of QAHAMAs used in the addition reaction with TMDI (formation of QAUDMAs).

Reagent	Mass (g)
QAHAMA-8	40.66
QAHAMA-10	43.66
QAHAMA-12	46.67
QAHAMA-14	49.67
QAHAMA-16	52.67
QAHAMA-18	55.67

## Data Availability

Data supporting reported results are available from the authors.

## References

[1] James S.L., Abate D., Abate K.H., Abay S.M., Abbafati C., Abbasi N., Abbastabar H., Abd-Allah F., Abdela J., Abdalalim A. (2018). Global, regional, and national incidence, prevalence, and years lived with disability for 354 Diseases and Injuries for 195 countries and territories, 1990–2017: A systematic analysis for the Global Burden of Disease Study 2017. Lancet.

[2] Li X., Kolltveit K.M., Tronstad L., Olsen I. (2000). Systemic diseases caused by oral infection. Clin. Microbiol. Rev..

[3] Lu M., Xuan S., Wang Z. (2019). Oral microbiota: A new view of body health. Food Sci. Hum. Wellness.

[4] Pratap B., Gupta R.K., Bhardwaj B., Nag M. (2019). Resin based restorative dental materials: Characteristics and future perspectives. Jpn. Dent. Sci. Rev..

[5] Beyth N., Yudovin-Farber I., Basu A., Weiss E.I., Domb A.J., Subramani K., Ahmed W. (2018). Antimicrobial Nanoparticles in Restorative Composites. Emerging Nanotechnologies in Dentistry.

[6] Irie M., Suzuki K., Watts D.C. (2002). Marginal gap formation of light-activated restorative materials: Effects of immediate setting shrinkage and bond strength. Dent. Mater..

[7] Chrószcz M.W., Barszczewska-Rybarek I.M. (2020). Nanoparticles of quaternary ammonium polyethylenimine derivatives for application in dental materials. Polymers.

[8] Song W., Ge S. (2019). Application of antimicrobial nanoparticles in dentistry. Molecules.

[9] Xiao Y.H., Chen J.H., Fang M., Xing X.D., Wang H., Wang Y.J., Li F. (2008). Antibacterial effects of three experimental quaternary ammonium salt (QAS) monomers on bacteria associated with oral infections. J. Oral Sci..

[10] Gilbert P., Moore L.E. (2005). Cationic antiseptics: Diversity of action under a common epithet. J. Appl. Microbiol..

[11] Makvandi P., Jamaledin R., Jabbari M., Nikfarjam N., Borzacchiello A. (2018). Antibacterial quaternary ammonium compounds in dental materials: A systematic review. Dent. Mater..

[12] Zhang Y., Chen Y., Hu Y., Huang F., Xiao Y. (2018). Quaternary ammonium compounds in dental restorative materials. Dent. Mater. J..

[13] Xue Y., Xiao H., Zhang Y. (2015). Antimicrobial polymeric materials with quaternary ammonium and phosphonium salts. Int. J. Mol. Sci..

[14] Ebi N., Imazato S., Noiri Y., Ebisu S. (2001). Inhibitory effects of resin composite containing bactericide-immobilized filler on plaque accumulation. Dent. Mater..

[15] Vidal M.L., Rego G.F., Viana G.M., Cabral L.M., Souza J.P.B., Silikas N., Schneider L.F., Cavalcante L.M. (2018). Physical and chemical properties of model composites containing quaternary ammonium methacrylates. Dent. Mater..

[16] Makvandi P., Ghaemy M., Mohseni M. (2016). Synthesis and characterization of photo-curable bis-quaternary ammonium dimethacrylate with antimicrobial activity for dental restoration materials. Eur. Polym. J..

[17] Chai Z., Li F., Fang M., Wang Y., Ma S., Xiao Y., Huang L., Chen J. (2011). The bonding property and cytotoxicity of a dental adhesive incorporating a new antibacterial monomer. J. Oral Rehabil..

[18] Huang L., Xiao Y.H., Xing X.D., Li F., Ma S., Qi L.L., Chen J.H. (2011). Antibacterial activity and cytotoxicity of two novel cross-linking antibacterial monomers on oral pathogens. Arch. Oral Biol..

[19] Antonucci J.M., Zeiger D.N., Tang K., Lin-Gibson S., Fowler B.O., Lin N.J. (2012). Synthesis and characterization of dimethacrylates containing quaternary ammonium functionalities for dental applications. Dent. Mater..

[20] Manouchehri F., Sadeghi B., Najafi F., Mosslemin M.H., Niakan M. (2019). Synthesis and characterization of novel polymerizable bis-quaternary ammonium dimethacrylate monomers with antibacterial activity as an efficient adhesive system for dental restoration. Polym. Bull..

[21] Huang L., Yu F., Sun X., Dong Y., Lin P.T., Yu H.H., Xiao Y.H., Chai Z.G., Xing X.D., Chen J.H. (2016). Antibacterial activity of a modified unfilled resin containing a novel polymerizable quaternary ammonium salt MAE-HB. Sci. Rep..

[22] Liang X., Sӧderling E., Liu F., He J., Lassila L.V.J., Vallittu P.K. (2014). Optimizing the concentration of quaternary ammonium dimethacrylate monomer in bis-GMA/TEGDMA dental resin system for antibacterial activity and mechanical properties. J. Mater. Sci. Mater. Med..

[23] Liang X., Huang Q., Liu F., He J., Lin Z. (2013). Synthesis of novel antibacterial monomers (UDMQA) and their potential application in dental resin. J. Appl. Polym. Sci..

[24] Huang Q., Lin Z., Liang X., Liu F., He J. (2014). Preparation and characterization of antibacterial dental resin with UDMQA-12. Adv. Polym. Technol..

[25] Okeke U.C., Snyder C.R., Frukhtbeyn S.A. (2019). Synthesis, purification and characterization of polymerizable multifunctional quaternary ammonium compounds. Molecules.

[26] Li J.W., Lee H.T., Tsai H.A., Suen M.C., Chiu C.W. (2018). Synthesis and properties of novel polyurethanes containing long-segment fluorinated chain extenders. Polymers.

[27] Sideridou I., Tserki V., Papanastasiou G. (2002). Effect of chemical structure on degree of conversion in light-cured dimethacrylate-based dental resins. Biomaterials.

[28] Barszczewska-Rybarek I.M. (2019). A guide through the dental dimethacrylate polymer network structural characterization and interpretation of physico-mechanical properties. Materials.

[29] Manappallil J.J. (2015). Basic Dental Materials.

[30] Charton C., Falk V., Marchal P., Pla F., Colon P. (2007). Influence of Tg, viscosity and chemical structure of monomers on shrinkage stress in light-cured dimethacrylate-based dental resins. Dent. Mater..

[31] Brandrup J., Immergut E.H., Grulke E.A. (1999). Polymer Handbook.

[32] Barszczewska-Rybarek I.M., Chrószcz M.W., Chladek G. (2021). Physicochemical and mechanical properties of bis-gma/tegdma dental composite resins enriched with quaternary ammonium polyethylenimine nanoparticles. Materials.

[33] Watts D.C., Ratner B.D., Hoffman A.S., Schoen F.J., Lemons J.E. (2013). Adhesive and Sealants. Biomaterials Science: An Introduction to Materials.

[34] ISO 489:1999 (1999). Plastics—Determination of Refractive Index.

